# Supplementary material for: Explicitly nonlinear fMRI networks reveal hidden trajectories of infant brain development
Source: bioRxiv. 2026 Apr 7:2026.04.07.716703. Preprint. [Version 1] doi: 10.64898/2026.04.07.716703 (PMC13082082; doi:10.64898/2026.04.07.716703)
Supplement: Supplement 1 [file media-1.pdf]

Supplementary Information for Kinsey et al. “Explicitly nonlinear networks reveal hidden trajectories of infant brain development”

Table S1. Sample demographic information. For demographic categories with a participant number less than the total sample, *n* is specified in the category title. SD: standard deviation. rsfMRI: resting-state functional magnetic resonance imaging. GED: general educational development.

**Sex (no.)**	**Race (no.),** ***n*** **= 64**	**Maternal education (no.),** ***n*** **= 62**	**Household income in United States dollars (no.),** ***n*** **= 61**	**Gestational age at birth in weeks (mean ± SD)**	**No. rsfMRI scans**
Male (42)	White (33)<br>Black or African American (3)<br>More than one race (1)	High school or GED (1)<br>Trade/vocational training (0)<br>College coursework (1)<br>Associate’s degree (1)<br>College degree (13)<br>Master’s degree (16)<br>Professional degree (2)	< $40000 (1)<br>$40000-$60000 (5)<br>$60001-$80000 (2)<br>$80001-$100000 (7)<br>$100001-$125000 (6)<br>$125001-$150000 (4)<br>> $150000 (9)	39.17 ± 1.46	78
Female (30)	White (23)<br>Black or African American (2)<br>More than one race (2)	High school or GED (0)<br>Trade/vocational training (1)<br>College coursework (3)<br>Associate’s degree (0)<br>College degree (9)<br>Master’s degree (11)<br>Professional degree (4)	< $40000 (2)<br>$40000-$60000 (2)<br>$60001-$80000 (3)<br>$80001-$100000 (7)<br>$100001-$125000 (4)<br>$125001-$150000 (4)<br>> $150000 (5)	39.18 ± 1.16	52

Table S2. Summary information for assessment of spatial variation between concordant explicitly nonlinear (ENL) and linear (LIN) intrinsic connectivity network (ICN) counterparts. Statistical tests were voxel-wise two-sided paired samples *t*-tests. Confidence interval (CI) information is reported as mean ± std CI range, while summary effect size information is reported as Cohen's *D* mean ± standard deviation (SD). rsfMRI: resting-state functional magnetic resonance imaging. SUB: subcortical. CER: cerebellum. VIS1: primary visual. TEMP1: primary temporal. MTR1: primary sensorimotor. VIS2: secondary visual. TEMP2: secondary temporal. MTR2: secondary sensorimotor. VIS3: tertiary visual. ATN: dorsal attention. TRI: triangularis. FRON: frontal. aDM: anterior default mode. pDM: posterior default mode.

**ICN**	***n* (rsfMRI scans)/*df***	**CI range (mean ± std) - ENL > LIN**	**CI range (mean ± SD) - LIN > ENL**	**Cohen's *D* (mean ± SD) ENL > LIN**	**Cohen's *D* (mean ± SD) LIN > ENL**
SUB	130/129	0.3250 ± 0.0303	0.3602 ± 0.0311	0.4790 ± 0.1934	0.5533 ± 0.2141
CER	130/129	0.4211 ± 0.0365	0.3894 ± 0.0457	0.2201 ± 0.0175	0.3516 ± 0.1145
VIS1	130/129	0.3481 ± 0.0286	0.3781 ± 0.0340	0.2430 ± 0.0440	0.5083 ± 0.1886
TEMP1	130/129	0.3671 ± 0.0306	0.3990 ± 0.0415	0.2819 ± 0.0689	0.6017 ± 0.2361
MTR1	130/129	0.4049 ± 0.0356	0.3534 ± 0.0442	0.4188 ± 0.1749	0.5708 ± 0.2931
VIS2	130/129	0.4303 ± 0.0343	0.4031 ± 0.0477	0.4911 ± 0.1846	0.6326 ± 0.2405
TEMP2	130/129	0.3479 ± 0.0358	0.3331 ± 0.0322	0.6265 ± 0.2035	0.5895 ± 0.2960
MTR2	130/129	0.3690 ± 0.0371	0.3671 ± 0.0425	0.4254 ± 0.1709	0.5016 ± 0.2081
VIS3	130/129	0.4122 ± 0.0357	0.3695 ± 0.0320	0.8121 ± 0.3376	1.1214 ± 0.5010
ATN	130/129	0.3937 ± 0.0351	0.3522 ± 0.0431	0.4601 ± 0.1595	0.6030 ± 0.3036

TRI	130/129	0.3260 ± 0.0290	0.3229 ± 0.0273	0.5333 ± 0.2178	0.6506 ± 0.3045
FRON	130/129	0.3514 ± 0.0291	0.3378 ± 0.0400	0.5861 ± 0.2443	0.5601 ± 0.2512
aDM	130/129	0.3515 ± 0.0341	0.3419 ± 0.0303	0.7082 ± 0.3729	0.6595 ± 0.2705
pDM	130/129	0.3410 ± 0.0288	0.3248 ± 0.0304	0.3575 ± 0.1241	0.7156 ± 0.3250

Table S3. Summary information for intrinsic connectivity network (ICN) associations with age. Statistical tests were generalized additive model *F* tests. Summary model effective degrees of freedom (EDF; quantifies nonlinearity of developmental trajectory) information for all tested voxels is reported as mean ± standard deviation (SD). ENL: explicitly nonlinear. LIN: linear. rsfMRI: resting-state functional magnetic resonance imaging. SUB: subcortical. CER: cerebellum. VIS1: primary visual. TEMP1: primary temporal. MTR1: primary sensorimotor. VIS2: secondary visual. TEMP2: secondary temporal. MTR2: secondary sensorimotor. VIS3: tertiary visual. ATN: dorsal attention. TRI: triangularis. FRON: frontal. aDM: anterior default mode. pDM: posterior default mode. VIS4: quaternary visual. lFP: left frontoparietal. rFP: right frontoparietal. PREF: prefrontal. SAL: salience. lPCG: left postcentral gyrus. lTPO: left temporo-parietal-occipital.

**ICN**	**No. significant voxels - ENL**	**No. significant voxels - LIN**	***n* (rsfMRI scans)**	**EDF (mean ± SD) - ENL**	**EDF (mean ± SD) - LIN**	**No. voxels EDF ≥ 3 - ENL**	**No. voxels EDF ≥ 3 - LIN**
SUB	835	1014	130	1.6557 ± 1.0177	2.0388 ± 0.8054	99	95
CER	1239	1139	130	1.7182 ± 0.7207	2.0621 ± 0.9227	50	188
VIS1	1748	1644	130	2.0497 ± 0.8631	1.8566 ± 0.8963	199	206
TEMP1	1606	1634	130	1.5728 ± 0.8672	2.3411 ± 1.1326	157	483
MTR1	1941	480	130	1.6753 ± 0.7890	1.2716 ± 0.5487	125	8
VIS2	16	1562	130	1.5199 ± 0.8462	1.4767 ± 0.8190	11	101
TEMP2	1662	605	130	1.5199 ± 0.8462	1.9103 ± 1.0353	137	85
MTR2	1192	1801	130	1.9613 ± 0.8337	2.0487 ± 1.0160	117	310
VIS3	206	356	130	1.6937 ± 1.1964	2.4646 ± 1.6593	23	115
ATN	1404	218	130	2.3340 ± 1.5716	1.7044 ± 0.9724	446	24
TRI	1285	1145	130	2.6817 ± 1.0842	2.1486 ± 0.9642	589	225
FRON	1507	1379	130	1.9833 ± 0.9875	1.8629 ± 0.8358	195	119

aDM	2279	871	130	1.6739 ± 0.7842	1.4732 ± 0.6637	120	17
pDM	386	295	130	2.1205 ± 1.0371	1.7691 ± 0.9698	86	29
VIS4	6	-	130	3.1397 ± 0.7795	-	4	-
lFP	0	-	130	-	-	0	-
rFP	243	-	130	2.2077 ± 1.2804	-	74	-
PREF	835	-	130	1.7501 ± 1.1249	-	128	-
SAL	621	-	130	2.2390 ± 1.0813	-	141	-
lPCG	-	1357	130	-	1.7284 ± 0.9542	-	125
lTPO	-	723	130	-	1.6468 ± 1.0058	-	39
Total	19011	16223	-	-	-	2701	2169

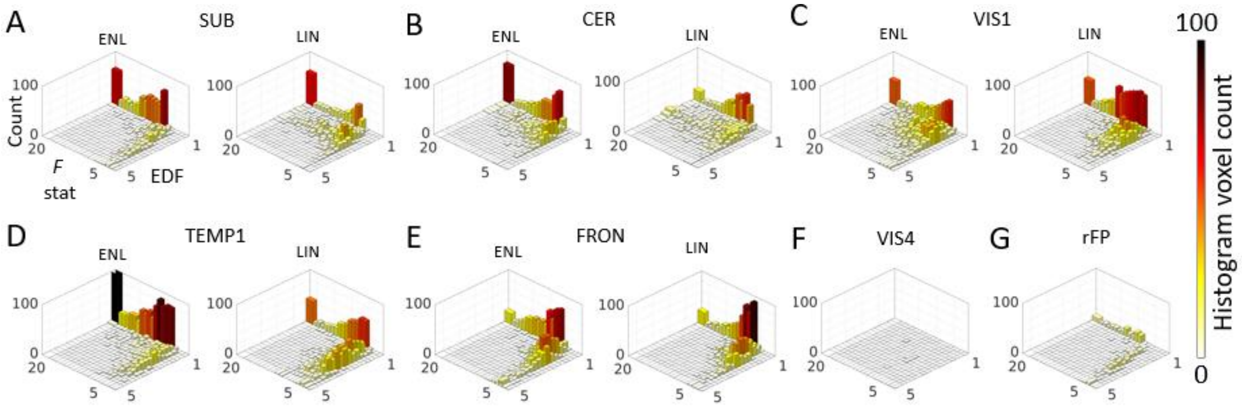

Fig. S1. Geometric complexity of explicitly nonlinear (ENL) and linear (LIN) intrinsic connectivity network developmental trajectories. (A-G) Two-dimensional histograms depict age association *F* statistics against effective degrees of freedom (EDF, quantifies nonlinearity of developmental trajectory) for significant ENL and LIN voxels for a subset of networks. Hue and bar height represent voxel count. SUB: subcortical. CER: cerebellum. VIS1: primary visual. TEMP1: primary temporal. FRON: frontal. VIS4: quaternary visual (ENL). rFP: right frontoparietal (ENL).

Table S4. Results of generalized additive model effective degrees of freedom (EDF, quantifies nonlinearity of developmental trajectory) comparisons between significant explicitly nonlinear (ENL) and linear (LIN) intrinsic connectivity network (ICN) voxels. Statistical tests were two-sided independent samples permutation tests with 5000 random permutations. Effect size is reported as Hedges's *g*. SUB: subcortical. CER: cerebellum. VIS1: primary visual. TEMP1: primary temporal. MTR1: primary sensorimotor. VIS2: secondary visual. TEMP2: secondary temporal. MTR2: secondary sensorimotor. VIS3: tertiary visual. ATN: dorsal attention. TRI: triangularis. FRON: frontal. aDM: anterior default mode. pDM: posterior default mode.

**ICN**	***n* (significant ENL voxels)**	***n* (significant LIN voxels)**	**EDF difference (ENL – LIN)**	***p***	**Hedges's *g***
SUB	835	1014	–0.3831	< .001	0.4222
CER	1239	1139	–0.3439	< .001	0.4175
VIS1	1748	1644	0.1931	< .001	0.2195
TEMP1	1606	1634	–0.7683	< .001	0.7608
MTR1	1941	480	0.4037	< .001	0.5401
VIS2	16	1562	2.0974	< .001	2.5359
TEMP2	1662	605	–0.3903	< .001	0.4334
MTR2	1192	1801	−0.0874	.0154	-
VIS3	206	356	–0.7709	< .001	0.5117
ATN	1404	218	0.6296	< .001	0.4182
TRI	1285	1145	0.5331	< .001	0.5179
FRON	1507	1379	0.1204	< .001	0.1312
aDM	2279	871	0.2007	< .001	0.2666
pDM	386	295	0.3515	< .001	0.3485
Total	19011	16223	−0.0028	.8078	-

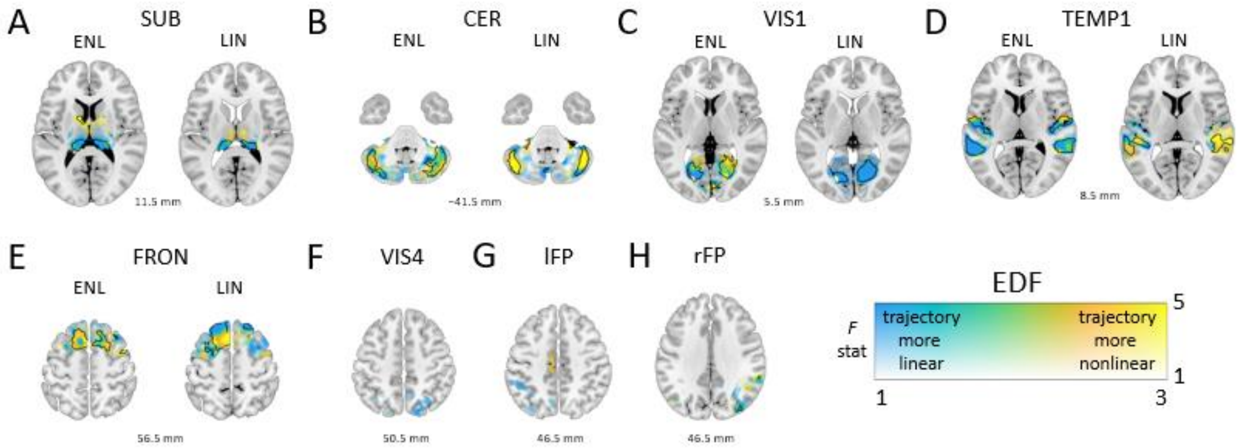

Fig. S2. Localization of explicitly nonlinear (ENL) and linear (LIN) intrinsic connectivity network associations with corrected postnatal age. (A-H) Spatial maps of voxel associations with age are plotted according to a dual-coded colormap for a subset of ENL and LIN networks, with transparency reflecting *F* statistic magnitude, hue representing model effective degrees of freedom (EDF; quantifies nonlinearity of developmental trajectory), and black contours indicating FDR-corrected significance ($q < .05$). Warmer hues indicate more complex, nonlinear developmental trajectories while cooler hues indicate less complex, approximately linear trajectories. Results are overlaid on the Montreal Neurological Institute 152 template with z coordinates listed relative to the origin. SUB: subcortical. CER: cerebellum. VIS1: primary visual. TEMP1: primary temporal. FRON: frontal. VIS4: quaternary visual (ENL). lFP: left frontoparietal (ENL). rFP: right frontoparietal (ENL).

Table S5. Results of comparisons between explicitly nonlinear (ENL) and linear (LIN) intrinsic connectivity network (ICN) statistical sensitivity to age. A two-sided McNemar's test was used to assess the overall ENL vs. LIN difference in statistical sensitivity (for all voxels tested across concordant ICNs), and differences in statistical sensitivity for concordant ICN pairs were assessed separately using two-sided McNemar's tests. Odds ratio (OR) is used as an indicator of effect size. SUB: subcortical. CER: cerebellum. VIS1: primary visual. TEMP1: primary temporal. MTR1: primary sensorimotor. VIS2: secondary visual. TEMP2: secondary temporal. MTR2: secondary sensorimotor. VIS3: tertiary visual. ATN: dorsal attention. TRI: triangularis. FRON: frontal. aDM: anterior default mode. pDM: posterior default mode.

ICN	No. significant voxels identified for ENL but not LIN	No. significant voxels identified for LIN but not ENL	*df*	χ^2	*p*	OR
SUB	280	492	1	57.6697	< .00001	1.76
CER	461	250	1	62.0253	< .00001	1.84
VIS1	524	555	1	0.8341	.3611	-
TEMP1	560	555	1	0.0143	.9046	-
MTR1	1397	143	1	1019.5	< .00001	9.77
VIS2	1	1380	1	1375	< .00001	1380
TEMP2	959	112	1	668.2689	< .00001	8.56
MTR2	111	467	1	218.0363	< .00001	4.21
VIS3	49	238	1	123.1498	< .00001	4.86
ATN	1199	37	1	1090.6	< .00001	32.41
TRI	469	477	1	0.0518	.82	-
FRON	597	654	1	2.5068	.1134	-
aDM	929	229	1	421.9352	< .00001	4.06
pDM	297	60	1	156.0112	< .00001	4.95
Total	7833	5649	1	353.4705	< .00001	1.39
